# History of the Organisation for Economic Co-operation and Development (OECD) test guidelines for non-animal test methods in Japan

**DOI:** 10.1186/s41021-024-00323-7

**Published:** 2025-01-29

**Authors:** Hajime Kojima

**Affiliations:** 1 Department of Pharmaceutical Engineering, Faculty of Engineering, Sanyo-Onoda City University, 1-1-1, Daigaku-dori, Sanyo-Onoda City, 756-0884 Yamaguchi Japan; 2 National Institute of Health Sciences, Kawasaki, Japan

**Keywords:** Organisation for Economic Co-operation and Development, Test guideline, Alternative test method, Validation

## Abstract

The number of alternatives to animal tests (non-animal test methods) for human health developed globally account for more than 40% of the test methods in the Organisation for Economic Co-operation and Development (OECD) Guidelines for the Testing of Chemicals (TGs). Within the TGs, the National Institute of Health Sciences (NIHS) has standardized 16 OECD TGs for human health, implemented four major revisions, and developed one test method for the International Council for Harmonization of Technical Requirements for Pharmaceuticals for Human Use (ICH) S10 guidelines on photosafety. This review describes trends in the OECD and Japan that mainly focus on international standardizations of non-animal test methods for human health. Drawing from this experience, I hope Japan will advance new approach methodologies for detecting systemic toxicity, which are in global demand.

## Background

Russel and Barch proposed the 3Rs principle in animal testing in 1959 [1]. Alternative testing refers to methods that reduce the number of animals used, refine procedures to minimize or eliminate pain or distress, enhance animal well-being, or replace animals with non-animal systems or non-sentient species, adhering to the 3Rs principle [2]. This review summarizes Japan’s contribution to the Organisation for Economic Co-operation and Development (OECD) relating to international standardization of non-animal test methods for human health.

### International development and validation of alternative methods

The development of non-animal testing methods commenced in the 1960s [3]. Initially, these methods were aimed at replacing the Draize eye or skin irritation tests and acute toxicity tests (lethal dose: LD_50_) [4]. Research and development of many alternative methods of eye irritation tests have been investigated [5, 6]. During the 1990s, large-scale validation studies of eye irritation tests [7, 8] and FRAME [9] were conducted by cosmetic industry associations in Europe and the United States. As an alternative to acute toxicity tests, collaborative research on cytotoxicity tests has been conducted, mainly in Sweden [10]. In Germany, the Centre for Documentation and Evaluation of Alternatives to Animal Experiments and the European Center for the Validation of Alternative Methods (ECVAM, the current name is EURL ECVAM) are leading the validation of alternative methods for corrosivity tests [11, 12]. Some of these validated methods have been adopted as OECD test guidelines (TG), providing harmonized methods for safety evaluation of chemical substances [13].

These test methods were established through validation studies that emphasize reproducibility and predictability. In particular, safety assessments require tests with high predictability; therefore, protocols with higher sensitivity than animal test results (i.e., protocols that produce as few false negatives as possible) are required. During this validation process, protocols were refined, and test methods with high predictability within the scope of application were formalized. In the 1990s, this policy was ambiguous, and validation was performed at the discretion of individual groups. As a result of the lack of clarity in validation requirements, many test methods were either not proposed to the OECD or abandoned during the OECD review process.

### International trends in the validation and adoption of non-animal test methods

To streamline validated methods for regulatory acceptance, the OECD held the Solna Workshop on the Harmonization of Validation and Acceptance Criteria for Alternative Toxicological Test Methods in 1996 with experts from various countries. The principles and criteria for validation and regulatory acceptance of the new and revised test methods were agreed upon in this workshop. Additionally, the OECD Conference on “Validation and Regulatory Acceptance of New and Updated Methods in Hazard Assessment” was held in Stockholm, Sweden, in March 2002. The purpose of the conference was to develop and achieve consensus on practical guidance for the principles and processes for the validation and acceptance of animal and non-animal test methods for regulatory hazard assessment purposes. Based on this conference, OECD GD No. 34, “The Validation and International Acceptance of New or Updated Test Methods for Hazard Assessment,” was published in 2005 [2]. This document provides guidance on issues related to validating new or updated test methods that are consistent with current approaches. Test method validation is a process based on scientifically sound principles through which the reliability and relevance of a particular test, approach, method, or process are established for a specific purpose.

Before this guidance, various validation centers were established worldwide. The National Toxicology Program Interagency Center for the Evaluation of Alternative Toxicological Methods and Interagency Coordinating Committee on the Validation of Alternative Methods (ICCVAM) [14] was established in the United States in 1991. ECVAM [15] was established in Europe in 1997. These centers operate under respective laws and focus on developing and evaluating alternative methods. In Japan, the Japanese Centre for the Validation of Alternative Methods (JaCVAM) was established within the National Institute of Health Sciences (NIHS) in 2005 [16]. To avoid redundant studies and accelerate research, the International Cooperation on Alternative Test Methods (ICATM) was established by ICCVAM, ECVAM, JaCVAM, and Health Canada in March 2009 [17]. The goal of the ICATM was to foster international co-operation in validation studies, independent peer review, and harmonized recommendations, ensuring worldwide acceptance of alternative methods and strategies. These test methods/strategies were developed with the aim to provide equivalent or improved protection for people, animals, and the environment, while replacing, reducing, or refining animal use where scientifically feasible. As the importance of international co-operation in the development of alternative methods has been emphasized, the Korean Centre for Alternative Methods and the Brazilian Centre for Alternative Methods were established, and subsequently joined this organization [18, 19].

Since the 1980s, the OECD has adopted in vitro tests for genotoxicity tests. However, since establishment of the other non-animal test methods have advanced substantially in 2004, the non-animal test methods have advanced over the past 20 years. This progress includes alternative methods to animal testing for corrosion, such as TG430: In vitro Skin Corrosion: Transcutaneous Electrical Resistance Test Method; TG 431: In vitro Skin Corrosion: Reconstructed human Epidermis Test Method; TG432: In vitro 3T3 Neutral Red Uptake Phototoxicity test; and TG 428: Skin Absorption, In vitro Method [12]. ICATM contributed substantially to the establishment of these TG.

As of November 2024, 31 *in chemico* or in vitro test methods have been adopted as alternative methods among 76 OECD TGs related to human health (Table 1) [13]. The number of non-animal test methods for human health developed globally exceeds 40% of the test methods adopted by OECD TGs. Table 2 lists the TGs for the non-animal test methods used in Japan. Each TG contains many similar test methods that are meant to me combined in toxicity evaluation. In total, there are 75 validated test methods that are included within the 31 TGs. Furthermore, good in vitro method practices have been developed to ensure the number and quality of in vitro studies [2]. However, the developed non-animal test methods are limited to skin irritation, phototoxicity, eye irritation, genotoxicity, and endocrine disruptor screening, contributing mainly to the United Nations globally harmonized system of classification and labeling of chemicals (UN GHS) [20]. Few methods for detecting systematic toxicity have been acceptedby the OECD.

**Table 1:** OECD guidelines for the testing of chemicals for health effects (2024)

Classification	Number of TGs	TGs for in vitro, *in chemico*
Skin Corrosion	3	3
Skin Irritation	2	1
Phototoxicity	3	3
Eye Irritation	10	9
Skin Sensitization	8	4
Skin Absorpution	2	1
Genotoxicity	13	5
Endocrine Disrupter	6	4
Others	29	1
Total	76	31

**Table 2:** Non-animal test methods approved by OECD TG

Classification	TG	Title	Names of Test method	Published	Remarks
Genotoxicity	471	Bacterial Reverse Mutation Test	Ames test	1983	
	472	*In vitro Mammalian Chromosomal Aberration Test*		1983	
	476	In vitro Mammalian Cell Gene Mutation Tests using the Hprt and xprt genes		1984	
	487	*In vitro Mammalian Cell Micronucleus Test*		2010	
	490	In vitro Mammalian Cell Gene Mutation Tests Using the Thymidine Kinase Gene		2015	
Skin Corrosion	430	*In vitro Skin Corrosion: Transcutaneous Electrical Resistance Test Method (TER)*		2004	
	431	*In vitro Skin Corrosion: Reconstructed Human Epidermis (RHE) Test Method*	EpiSkin™(VRM)	2004	
			EpiDerm™ SIT (EPI200)(VRM)	2004	
			SkinEthic™ RHE	2004	
			epiCS^®^	2016	
			LabCyte EPI-MODEL24 SCT	2019	JaCVAM
	435	In vitro Membrane Barrier Test Method for Skin Corrosion	CORROSITEX	2006	
Skin Irritation	439	*In vitro Skin Irritation: Reconstructed human Epidermis Test Method*	EpiSkin™(VRM)	2010	
			EpiDerm™ SIT (EPI200)(VRM)	2010	
			SkinEthic™ RHE	2010	
			LabCyte EPI-MODEL24 SIT	2013	JaCVAM
			epiCS^®^	2019	
			Skin+^®^	2019	
			KeraSkin™ SIT	2021	
Serious Eye Damage and Not Requiring Classification for Eye Irritation or Serious Eye Damage	437	Bovine Corneal Opacity and Permeability Test Method for Identifying (i) Chemicals Inducing Serious Eye Damage and (ii) Chemicals Not Requiring Classification for Eye Irritation or Serious Eye Damage	Bovine Corneal Opacity and Permeability Test Method	2009	
	438	Isolated Chicken Eye Test Method for Identifying (i) Chemicals Inducing Serious Eye Damage and (ii) Chemicals Not Requiring Classification for Eye Irritation or Serious Eye Damage	Isolated Chicken Eye Test Method	2009	
	460	Fluorescein Leakage Test Method for Identifying Ocular Corrosives and Severe Irritants	Fluorescein Leakage Test Method	2012	
	491	Short Time Exposure In vitro Test Method for Identifying (i) Chemicals Inducing Serious Eye Damage and (ii) Chemicals Not Requiring Classification for Eye Irritation or Serious Eye Damage	Short Time Exposure (STE) Test Method	2015	JaCVAM
	492	Reconstructed human Cornea-like Epithelium (RhCE) Test Method for Identifying Chemicals Not Requiring Classification and Labelling for Eye Irritation or Serious Eye Damage	EpiOcular™ EIT(OCL-200)	2017	
			SkinEthic™ HCE EIT(HCE/S)	2017	
			LabCyte CORNEA-MODEL24 EIT	2018	JaCVAM
			MCTT HCETM EIT	2024	
	492B	Reconstructed Human Cornea-like Epithelium (RhCE) Test Method for Eye Hazard Identification	SkinEthic™ RHE	2022	
	494	Vitrigel-Eye Irritancy Test Method for Identifying Chemicals Not Requiring Classification and Labelling for Eye Irritation or Serious Eye Damage	Vitrigel^®^-Eye Irritancy Test	2019	JaCVAM
	496	In vitro Macromolecular Test Method for Identifying Chemicals Inducing Serious Eye Damage and Chemicals Not Requiring Classification for Eye Irritation or Serious Eye Damage	Ocular Irritection (OI^®^)	2019	
			OptiSafe Eye Irritation Test™	2024	
	467	Defined Approaches for Serious Eye Damage and Eye Irritation		2022	
Skin Sensitisation	442 C	*In chemico* Skin Sensitisation assays addressing the Adverse Outcome Pathway key event on covalent binding to proteins	DPRA	2015	
			ADRA	2021	JaCVAM
			KDPRA	2022	
	442D	In vitro Skin Sensitisation assays addressing the Adverse Outcome Pathway Key Event on Keratinocyte activation	KeratinoSens	2015	
			LuSens	2022	
			EpiSensA	2024	JaCVAM
	442E	In vitro Skin Sensitisation assays addressing the Key Event on activation of dendritic cells on the Adverse Outcome Pathway for Skin Sensitisation	h-CLAT	2016	JaCVAM
			U-SENS™	2018	
			IL-8 Luc assay	2017	JaCVAM
			GARD	2023	
	497	Defined Approaches on Skin Sensitisation		2021	
Immunotoxicity	444 A	*In vitro Immunotoxicity*	IL-2 Luc assay	2023	JaCVAM
Phototoxicity	432	In vitro 3T3 NRU Phototoxicity Test	In vitro 3T3 NRU Test	2004	
	495	ROS (Reactive Oxygen Species) Assay for Photoreactivity	ROS assay	2019	JaCVAM
	498	In vitro Phototoxicity - Reconstructed human Epidermis Phototoxicity Test Method	EpiDerm™ (EPI-200)	2021	
Skin Absorption	428	Skin Absorption: In vitro Test Method		2004	
Endocrine Disrupter	455	Performance-Based Test Guideline for Stably Transfected Transactivation In vitro Assays to Detect Estrogen Receptor Agonists and Antagonists	STTA	2016	JaCVAM
			VM7Luc ER TA assay	2016	JaCVAM
	456	H295R Steroidogenesis Assay		2011	
	457	BG1Luc Estrogen Receptor Transactivation Test Method for Identifying Estrogen Receptor Agonists and Antagonists		2012	Deleted on 29 January 2018
	458	Stably Transfected Human Androgen Receptor Transcriptional Activation Assay for Detection of Androgenic Agonist and Antagonist Activity of Chemicals	AR-EcoScreen™	2016	Japan
			AR-CALUX^®^	2020	
			ARTA method using the 22Rv1/MMTV_GR-KO cell line	2023	
	493	Performance-Based Test Guideline for Human Recombinant Estrogen Receptor (hrER) In vitro Assays to Detect Chemicals with ER Binding Affinity	The Freyberger-Wilson (FW) In vitro Estrogen Receptor (ER) Binding Assay Using a Full Length Human Recombinant Erα	2018	
			The Chemical Evaluation and Research Institute (CERI) In vitro Estrogen Receptor Binding Assay Using a Human Recombinant Ligand Binding Domain Protein	2018	Japan

JaCVAM: The test methods which were validated by JaCVAM, Japan: The test methods which were approved by Japanese test develpers.

These alternative methods are useful for the hazard identification of chemical substances as required by the UN GHS classification; however, their applicability domain is limited. They cannot be used for comprehensive risk assessment ofchemical substances. For example, the non-animal test method TGs for skin sensitization and eye irritation can not be used on their own to evaluate these endpoints under the UN GHS. However, Guideline 497 was issued in 2021 to describe a defined approach that integrates non-animal TGs for skin sensitization, enabling UN GHS classification (1 A: strong, 1B: weak, non-category). Similarly, TG467 on the defined approach for serious eye damage and eye irritation allows the classification of weak irritation (UN GHS category 2) by combining tests and enabling in silico evaluation for non-eye irritants not classified under UN GHS [13]. Commercial software and OECD toolboxes are available for in silico applications [21].

### History of official non-animal test method formulation in Japan

In Japan, Dr. Yasuo Ohno (NIHS), his colleagues, and members of the Japan Cosmetic Industry Association conducted a validation study of alternative methods for eye irritation, which was funded by the Health Sciences Research program of the Ministry of Health, Labour and Welfare (MHLW) [22]. This study was the largest validation study conducted in Japan. It was meticulously planned and included animal experiments. However, the study focused on cosmetic raw materials, lacked independent peer review after validation, and was not accepted by MHLW, leading to the establishment of JaCVAM.

The Japanese Society of Alternative Methods for Animal Experiments (JSAAE) sponsored a validation study led by Dr. Tadao Ohno (RIKEN BioResource Research Center) and his colleagues, focusing on alternative methods for testing eye irritation [23]. This collaborative study involved over 50 participating institutions. Several studies presented cytotoxicity tests, which were meaningful from the viewpoint of dissemination of the test methods; however, the data sheet was incomplete, and biostatistical analysis took over a year. These challenges informed improvements in subsequent validation studies.

Within the JSAAE, collaborative studies on skin irritation models and alternative methods for eye irritation tests were conducted. A validation committee was established in response to the growing momentum to establish rules for collaborative studies. Various validation studies have been conducted to establish alternatives to skin corrosion tests [24]. Validation studies for two skin sensitization tests, led by JSAAE, were conducted for the Local Lymph Node Assay (LLNA)-Daicel (DA) developed by Daicel Corporation and the LLNA: BrdU-ELISA developed by the Chemicals Evaluation and Research Institute, officially established by the OECD in 2010 [25, 26]. These validation results were independently reviewed by ICCVAM, which led to the establishment of OECD TG442A: 442 A: Skin Sensitization, LLNA: DA and TG442B: Skin Sensitization, LLNA: BrdU-ELISA [12]. Without these validation studies, the international debut of JaCVAM and Japan would have been delayed by several years. Moreover, the establishment of ICATM, supported by validation centers in the United States and Europe, might not have occurred.

JaCVAM was established in November 2005 to promote alternative methods to animal testing in regulatory studies, adhering to the 3Rs principle wherever possible. It also fulfills the responsibility of the Center for Biological Safety and Research in NIHS to protect the general public by assessing the safety of chemicals and other materials, as stipulated in the NIHS regulations. JaCVAM activities are also beneficial for the application and approval of the manufacture and sale of pharmaceuticals and other products and contribute to the revision of cosmetic product standards. To achieve this, JaCVAM assesses the utility, limitations, and suitability of test methods for regulatory studies to determine the safety of chemicals and other materials and contributes to validation studies when necessary. Additionally, JaCVAM cooperates and collaborates with similar organizations in related fields in Japan and internationally.

As part of its mission for validation through international co-operation, JaCVAM evaluates test methods developed in Japan through validation studies of test methods and independent peer review. This work has led to the development of over 20 validation reports and peer-reviewed reports, including those developed by other countries or the OECD and supported by ICATM.

Japanese scientific societies also supports international validation studies conducted via JaCVAM. The MMS Study Group under the Japanese Environmental Mutagenesis Society (currently the Japanese Environmental Mutagen and Genom Society), together with ECVAM, ICCVAM, and the International Working Group for Genotoxicity, supported various activities. With contributions from Drs. Makoto Hayashi (NIHS) and Yoshifumi Uno (Mitsubishi Tanabe Pharma Cooperation) and international institutional collaboration, the protocol and validation report for the comet assay focused on the stomach and intestine were internationally agreed upon [2]. Consequently, TG489: In vivo mammalian alkaline comet assay was officially established in 2014 [13]. This process provided insights into organizing validation teams and creating validation reports.

Successful international peer reviews rely on support from ICCVAM. Thanks to ICCVAM peer reviews of the LLNA-DA and LLNA: BrdU-ELISA and the Short Time Exposure in vitro Test Method, JaCVAM was able to submit these reports to the OECD. The OECD approved these assays as TG442A, TG442B and TG491, and the experience provided knowledge on organizing international peer review accepted by the OECD.

Test developers of alternative methods are key players prior to method validation. As a first case validated non-animal test method by JaCVAM, J-TEC Corporation made significant contributions in Japan. The LabCyte EPI-MODEL24 SIT (Skin Irritation Test) validation studies weresupported by J-TEC Corporation. This test is described in TG439, In vitro Skin Irritation: Reconstructed human Epidermis (RhE) Test Method. EPI-MODEL24 SCT (Skin Corrosion Test) was also validated and approved in TG431, In vitro Skin Corrosion: Reconstructed human Epidermis (RhE) Test Method.

Numerous test developers have contributed to eye irritation testing. For example, TG491: Short Time Exposure in vitro Test Method was developed by the Kao Corporation. J-TEC Corporation developed Test No. 492: Reconstructed human Cornea-like Epithelium Test Method to identify chemicals that do not require classification and labeling for eye irritation or serious eye damage. LabCyte CORNEA-MODEL24 EIT popularized the use of three-dimensional models in Japan. Dr. TG494: The Vitrigel^®^-Eye Irritancy Test Method for identifying chemicals not requiring classification and labelling for eye irritation or serious eye damage, developed by Toshikaki Takezawa (The National Agricultural Research Organization) with Kanto Chemical Co., Inc., evaluates eye irritation over a short time period; the market for these tests is gradually expanding [13].

Furthermore, alternative skin sensitization testing methods have been developed in Japan. Starting with the h-CLAT assay developed by Kao Corporation and Shiseido Co., Ltd., various *in chemico* or in vitro Skin Sensitization tests, such as IL-8 Luc assay, ADRA(the Amino Acid Derivative Reactivity Assay), and EpisensA were included in TG442C to TG442E, thereby increasing Japan’s international presence in this field [13]. Based on these foundations and the contribution of Drs. Setsuya Aiba and Yutaka Kimura (Tohoku University), the world’s first non-animal immunotoxicity assay was introduced: TG444A In vitro immunotoxicity IL-2 Luc assay [13].

Efforts involving NIHS experts and test developers have formalized: 16 OECD TGs including animal test methods (Table 3), four major revisions to TGs related to human health [13], and the International Council for Harmonization of Technical Requirements for Pharmaceuticals for Human Use (ICH) guideline (S10 guideline on Photosafety with Reactive Oxygen Species assay) [26]. The latter assay is now approved in the TG program. The results, including validation reports and independent peer review reports related to TGs, have been published on the OECD and JaCVAM websites [16].

Unfortunately, the Bhas 42 Cell Transformation Assay could not be converted into a TG. Based on recommendations from Dr. Noriho Tanaka (Hatano Research Institute, Food and Drug Safety Center) and colleagues, JaCVAM participated in the international validation of this assay; however, the results were insufficient to support the development of a TG for this method. The assay remains limited to the OECD Series on Testing & Assessment No. 231, the Guidance Document on the In vitro Bhas 42 Cell Transformation Assay [2]. Dr. Kiyomi Omori (Kanagawa Prefectural Institute of Public Health) and colleagues are reattempting to establish a TG for this assay.

**Table 3:** Animal test methods in OECD TG developed by Japan

TG	Title	Published	Remarks
441	Uterotrophic Bioassay in Rodents A Short-Term Screening Test for Oestrogenic Properties	2007	
442 A	Skin Sensitization, Local Lymph Node Assay: DA	2010	JaCVAM
442B	Skin Sensitization, Local Lymph Node Assay: BrdU-ELISA or –FCM	2010	JaCVAM
489	In vivo Mammalian Alkaline Comet Assay	2016	JaCVAM

JaCVAM: The test methods which were validated by JaCVAM

The mission of JaCVAM is to evaluate the usefulness, limitations and suitability of test methods used for regulatory safety assessment of chemicals and other materials under the supervision of the JaCVAM Steering Committee. For this purpose, the JaCVAM Editorial Committee examines validation reports, peer review panel reports, and relevant background information, and shares these findings with related organizations worldwide. More than 40 JaCVAM-recommended test methods have been approved for regulatory use by the JaCVAM Regulatory Acceptance Board and they are submitted to MHLW.

Finally, the success of these 16 TGs cannot be solely attributed to JaCVAM members. Each validation study depended on the developers, while NIHS and international experts evaluated the results to ensure regulatory acceptance. Reflecting on each validation study, prioritizing the acceptance of Japanese TG within the OECD sometimes led to the dismissal of the developers’ perspective. Hence, the words and actions of international and OECD experts may have inadvertently offended some developers, because they have a concern that their published papers and its dataset on the test methods may be futile to be major modified the protocol by the validation studies.

On the other hand, needs for non-animal test methods are increasing from international organizations other than the OECD. JaCVAM is now conducting validation studies on skin irritation test methods with LabCyte EPI-MODEL 24 SIT (J-TEC Cooperation) for medical devices, MylcMAT, non-animal test methods for the pyrogenicity assay (MiCAN Technologies, Inc.) in Japanese Pharmacopeia.

Additionally, several validated methods, such as SIRC-CVS(Cristal Violet Staining) for eye irritation, the Bovine Corneal Opacity and Permeability Test Method with histopathological examination for eye irritation, Reconstructed human Skin Model, EPiTRI for in vitro skin irritation test, LbL-3D Skin SIT, Hand1 Luc EST (Embryo Stem cell Test) for Developmental and Reproductive Toxicity, and IL-1α Luc assay for in vitro Immunotoxicity Test, have yet to be accepted within the OECD TG program [12]. We apologize to the developers of these methods. Despite the challenges and failures mentioned above, these experiences should hopefully continue.

## Conclusion

In response to requests from international organizations other than the OECD, more methods from Japan will contribute to the development of new approach methodologies for systemic toxicity tests worldwide.

## Data Availability

Data that support the findings of this review have been publised in the OECD website and have no experiments on both animals and humans.

## References

[1] Russell WMS, Burch RL. The Principles of Humane Experimental Technique (1959).

[2] OECD Series on testing and assessment. (2024) https://www.oecd-ilibrary.org/environment/oecd-series-on-testing-and-assessment_20777876

[3] Weil CS, Scala RA. Study of intra- and interlaboratory variation in the results of rabbit eye and skin irritation test. Toxicol Appl Pharmcol. 1971;19:276–360. 10.1016/0041-008x(71)90112-8 5570968

[4] Balls M. The introduction and influence of the Concept of human experimental technique: editorials by balls. In: Comb M, R. and, Worth A, editors. The history of alternative test methods in Toxicology. Elsevier, London, UK: Academic; 2019.

[5] Editorials A, Sato T, Endo H. Toxicological alternative to animal tests,Eikodo,Tokyo(1993)(in Japanese).

[6] Editorials H, Ohno T. Manual of alternative to animal tests. KYORITSU SHUPPAN CO., LTD.,Tokyo(1994) (in Japanese).

[7] Gettings SD, et al. The CTFA Evaluation of Alternatives Program: an evaluation of in vitro alternatives to the Draize primary eye irritation test. (phase III) surfactant-based formulations. Food Chem Toxicol. 1996;34(1):79–117. 8603801 10.1016/0278-6915(96)89525-1

[8] Brantom PG, et al. A summary report of the COLIPA international validation study on alternatives to the draize rabbit eye irritation test. Toxicol Vitro. 1997;11(1–2):141–79. 10.1016/s0887-2333(96)00069-0 20654303

[9] ICCVAM: ICCVAM Recommended BCOP Test Method Protocol. In: ICCVAM test method evaluation report - in Vitro Ocular toxicity test methods for identifying ocular severe irritants and corrosives. Interagency Coordinating Committee on the validation of alternative methods (ICCVAM) and the National Toxicology Program (NTP) Interagency Center for the evaluation of alternative toxicological methods (NICEATM). NIH Publication No: 07–4517 (2006).

[10] Clemedson C, Kolman A, Forsby A. The integrated acute systemic toxicity project (ACuteTox) for the optimisation and validation of alternative in vitro tests. Altern Lab Anim. 2007;35(1):33–8. 17411349 10.1177/026119290703500102

[11] Spielmann H, et al. The International EU/COLIPA in Vitro Phototoxicity Validation Study: results of phase II (blind trial). Part 1: the 3T3 NRU phototoxicity test. Toxicol Vitro. 1998;12(3):305–27. 10.1016/s0887-2333(98)00006-x 20654413

[12] Fentem JH, Botham PA. ECVAM’s activities in validating alternative tests for skin corrosion and irritation. Altern Lab Anim. 2002;30(Suppl 2):61–7. 12572585 10.1177/026119290203002S09

[13] OECD Guidelines for the Testing of Chemicals. (2024) https://www.oecd-ilibrary.org/environment/oecd-guidelines-for-the-testing-of-chemicals_72d77764-en

[14] ICCVAM(. 2024) https://ntp.niehs.nih.gov/whatwestudy/niceatm/iccvam

[15] EURL ECVAM (2024) Available at: https://joint-research-centre.ec.europa.eu/reference-measurement/european-union-reference-laboratories/eu-reference-laboratory-alternatives-animal-testing-eurl-ecvam_en

[16] JaCVAM. (2024) Available at: http://jacvam.jp/.

[17] ICATM (2024) https://ntp.niehs.nih.gov/whatwestudy/niceatm/iccvam/international-partnerships/icatm

[18] KoCVAM (2024) https://www.nifds.go.kr/kocvamen/index.do

[19] Presgrave OA. Altern Lab Anim. 2008;36(6):705–8. 19154096 10.1177/026119290803600613

[20] UNECE, GHS Rev.10. (2023) https://unece.org/transport/documents/2023/07/standards/ghs-rev10

[21] OECD, QSAR tool box, http://www.qsartoolbox.org/(2024).

[22] Ono Y, et al. First phase inter-laboratory validation of the in vitro eye irritation test for cosmetic ingredinets. AATEX. 1995;3(4):121–209.

[23] Ohno T, et al. Validation study on five cytotoxicity assays by JSAAE. AATEX. 1998;5(1–2):1–86.

[24] Kojima H, et al. Validation of human skin models for skin corrosivity tests in Japan, Altern. Anim Test Exp. 2008;13(1):36–44.

[25] Omori T, et al. Interlaboratory validation of the modified murine local lymph node assay based on adenosine triphosphate measurement. J Pharmacol Toxicol Methods. 2008;58:11–26. 18593646 10.1016/j.vascn.2008.05.001

[26] Kojima H, et al. Inter-laboratory validation of the modified murine local lymph node assay based on 5-bromo-2’-deoxyuridine incorporation. J Appl Toxicol. 2011;31(1):63–74. 20677212 10.1002/jat.1567

